# Photon minibeam‐based LATTICE radiotherapy for small and medium‐sized tumors: A dosimetric planning study

**DOI:** 10.1002/mp.70596

**Published:** 2026-08-03

**Authors:** Wei Wu, Nimita Shinde, Jiaxin Li, Isabelle Vanhaezebrouck, Kai Jiang, Yuting Lin, Qiang Li, Hao Gao

**Affiliations:** ^1^ Institute of Modern Physics Chinese Academy of Sciences Lanzhou China; ^2^ University of Chinese Academy of Sciences Beijing China; ^3^ Department of Radiation Oncology University of Kansas Medical Center Kansas City USA; ^4^ Department of Radiation Oncology University of Texas Southwestern Medical Center Dallas USA

**Keywords:** LATTICE radiotherapy, Monte Carlo simulation, small and medium‐sized tumors, spatially fractionated radiotherapy

## Abstract

**Background:**

Lattice radiotherapy (LRT) is a spatially fractionated technique that delivers three‐dimensional high‐dose vertices within tumors. However, conventional LRT is constrained by geometric limitations when applied to small and medium‐sized tumor volumes, primarily due to the large beam sizes that restrict optimal lattice pattern formation.

**Purpose:**

This study aims to investigate a photon minibeam‐based LATTICE radiotherapy (mini‐LRT) approach designed to overcome the geometric limitations of conventional LRT and enable effective spatially fractionated treatment for small and medium‐sized tumors adjacent to critical organs.

**Methods:**

Three brain cases and three lung cases, with clinical target volumes ranging from 12.34 cc to 40.88 cc, were presented for treatment planning comparison between conventional LRT and mini‐LRT. A multi‐collimator delivery strategy employing shifted slit patterns was implemented to achieve complementary spatial coverage. Dose calculation and optimization were performed using Monte Carlo simulations and solved via an iterative convex relaxation algorithm. Reference stereotactic body radiation therapy (SBRT) plans were generated for all six cases, and setup‐error robustness analysis was performed for two representative cases using nominal and shifted dose‐influence matrix scenarios.

**Results:**

Mini‐LRT produced denser lattice structures in all cases, generating 5–11 vertices compared to only 1–3 vertices achievable with conventional LRT. Vertex diameters were reduced from 15.0 mm to 3.0–4.0 mm, and vertex‐to‐vertex distances decreased from 25.0 mm to 12.5 mm. The lattice volume ratio ranged from 0.68% to 1.07% with mini‐LRT. Organs at risk (OARs) dose reductions were observed in the evaluated cases, including reduced mean dose to brainstem, heart, and esophageal. Furthermore, mini‐LRT achieved a higher peak‐to‐valley dose ratio (PVDR) ranging from 2.19 to 2.94, compared to 1.75–2.34 for conventional LRT. Reference SBRT plans achieved clinical target volume (CTV) D_95_ = 50 Gy in all cases but showed elevated D_0.03cc_ to adjacent OARs in selected anatomically constrained cases. In the representative robustness analysis, worst‐case setup‐error scenarios showed modest PVDR degradation.

**Conclusions:**

This proof‐of‐concept dosimetric planning study suggests that photon minibeam‐based LATTICE radiotherapy can generate compact spatially fractionated dose distributions in selected small and medium‐sized tumors. Compared with conventional LRT, mini‐LRT increased vertex density, reduced lattice volume ratio, and improved OAR sparing in the evaluated cases. Additional experimental validation, motion assessment, and larger patient studies are required before clinical translation.

## 1 INTRODUCTION

Spatially Fractionated Radiotherapy (SFRT) is an advanced radiation treatment strategy that delivers heterogeneous dose distributions within target volumes, characterized by alternating regions of high‐ and low dose areas.^1^ This spatial modulation can enhance normal tissue sparing, maintain tumor control, and induce radiobiological responses, such as bystander effect and immune modulation.^2^ SFRT includes four main treatment modalities: GRID,^3^ LATTICE,^4^ minibeam ^5^ ^,^ and microbeam,^6^ each defined by its spatial scale and beam geometry. Among these, GRID and LATTICE have already been clinically implemented for the treatment of bulky or radioresistant tumors, showing promising tumor responses with reduced toxicity.^4^, ^7^, ^8^, ^9^, ^10^, ^11^, ^12^, ^13^

Lattice radiotherapy (LRT) delivers a 3D dose pattern with selectively positioned high‐dose vertices separated by predetermined distances, typically using intensity‐modulated radiation therapy (IMRT) or volumetric modulated arc therapy (VMAT).^4^, ^14^ The high‐dose vertices are generally 1–2 centimeters in diameter and separated by low‐dose valleys throughout the tumor volume.^10^, ^15^, ^16^ This centimeter‐scale spatial fractionation is suitable for treating large tumors, where sufficient intratumoral volume exists to accommodate distinct high‐ and low‐dose regions.^7^, ^17^, ^18^, ^19^ However, conventional LRT becomes geometrically constrained when applied to medium and small tumor volumes.^17^, ^18^, ^19^ Large vertex sizes and inter‐vertex spacing limit the formation of optimal lattice patterns, leading to either overlapping peaks or configurations with only one or two isolated high‐dose regions, thereby negating the biological benefits offered by spatial fractionation.

Currently, stereotactic radiosurgery (SRS) and stereotactic body radiation therapy (SBRT) represent the standard of care for small and medium‐sized tumors, offering excellent local control through the delivery of uniform, highly conformal ablative doses.^20^, ^21^, ^22^, ^23^, ^24^ However, the uniform high‐dose nature of SRS/SBRT becomes a critical limitation when tumors are near, or wrap around highly sensitive organs at risk (OARs), such as the brainstem, heart, or esophagus.^25^ In these geometrically constrained and high‐risk scenarios, delivering uniform ablative doses can lead to severe toxicities, such as radiation necrosis, cardiotoxicity, or esophageal fistula, often forcing clinicians to compromise by de‐escalating the prescribed dose.^26^, ^27^

Minibeam radiotherapy (MBRT) offers a potential solution to overcome this limitation. MBRT employs submillimeter‐wide and millimeter‐spacing beamlets that generate fine spatial dose heterogeneity, enabling precise treatment of smaller volumes while preserving tissue sparing.^5^, ^28^ This approach has been explored across multiple radiation modalities: proton MBRT ^29^, ^30^ and carbon ion MBRT,^31^, ^32^ electron MBRT ^33^ and, photon MBRT.^34^, ^35^ Preclinical studies in photon MBRT have demonstrated substantial normal tissue sparing and therapeutic gain. Several approaches have been developed attempting to broaden the clinical applications of SFRT treatment to smaller targets. Photon Mini‐Lattice ^36^ employs clinical beam sizes with 5 mm lattice vertices within targets. Proton scissor beam ^37^ utilizes standard clinical beam with optimization strategies focused on organs at risk (OARs) protection while maintaining uniform target dose delivery. Proton minibeam lattice ^38^ represents another attempt to use a smaller beam size to deliver a lattice.

Therefore, for small and medium‐sized tumors located adjacent to highly critical OARs, there is a need for a treatment paradigm that combines the ablative biological efficacy of SBRT with the normal tissue sparing capabilities of SFRT. To address this distinct clinical challenge, we propose a photon minibeam‐based LATTICE (mini‐LRT) approach. This approach can create smaller high‐dose vertices, increase vertex density, and preserve spatial dose modulation within limited target volumes. A multi‐collimator strategy is employed to achieve complementary dosimetric potential, and treatment planning is evaluated using Monte Carlo simulations and iterative convex relaxation optimization. Comparative planning analyses between mini‐LRT and conventional LRT are performed to assess vertex distribution characteristics and normal tissue sparing performance.

## 2 METHODS AND MATERIALS

### 2.1 Mini‐LRT

The proposed mini‐LRT approach uses photon minibeams to generate compact lattice‐like high‐dose vertices within small and medium‐sized tumors. Because megavoltage photon minibeams have relatively limited lateral scattering, the peak‐to‐valley dose modulation produced by a narrow‐slit collimator can be partially preserved at treatment depth.

**FIGURE 1:**
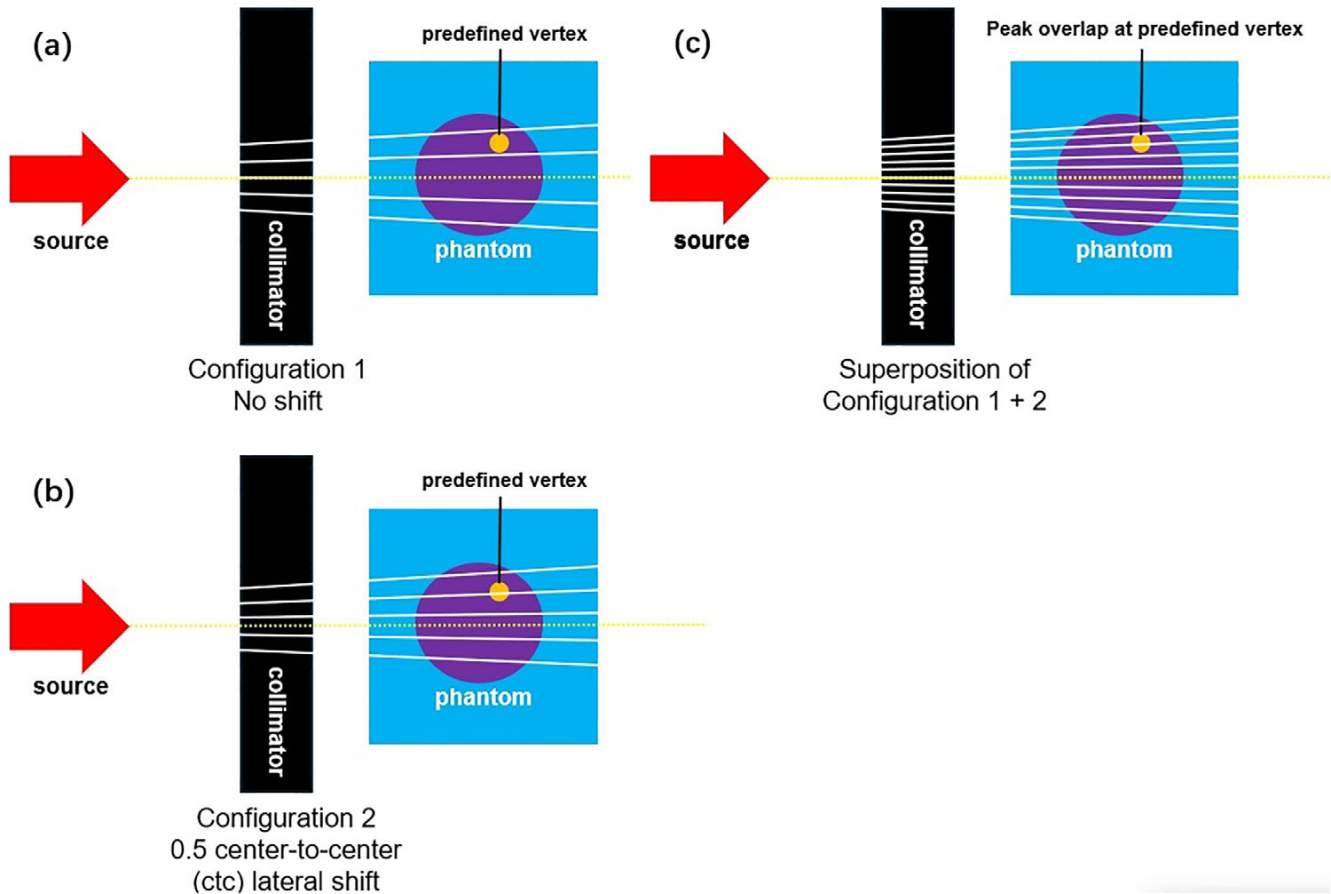
Schematic illustration of the shifted multi‐collimator mini‐LRT concept. (a) Configuration 1 (no shift) produces a periodic minibeam peak‐valley pattern, which may not align optimally with the predefined lattice vertex. (b) Configuration 2 applies a lateral 0.5 ctc shift, moving peak regions toward locations that are underdosed or blocked in Configuration 1. (c) Superposition of the two configurations improves peak coverage at the predefined vertex. Black: collimator structure; white: beam openings/minibeam peak regions; blue: phantom; yellow dashed line: central axis; purple: target; orange: predefined lattice vertex.

As illustrated in Figure 1(a), a fixed multi‐slit collimator produces a periodic peak‐valley pattern. In small targets, a predefined lattice vertex may not coincide with projected minibeam peak regions, so a single fixed slit configuration may provide insufficient peak accumulation at the desired vertex location. This geometric mismatch becomes more problematic when the target volume is limited and the lattice vertices are densely distributed.

To improve vertex coverage, we used multiple shifted collimator configurations. Each configuration had the same slit width and center‐to‐center (ctc) spacing but a different lateral offset, defined as 0, 0.25, 0.50, and 0.75 ctc. Therefore, regions located in valleys in one configuration could be irradiated by peaks in another configuration. By combining shifted slit patterns from multiple gantry angles, high‐dose peaks were preferentially accumulated at the predefined vertices while surrounding regions received lower valley doses.

By superimposing shifted slit patterns from multiple gantry angles and multiple collimator offsets, as illustrated in Figure 1(c), high‐dose peaks can be preferentially accumulated at the predefined lattice vertices, while the surrounding target volume remains at lower valley dose. This strategy improves the geometric compatibility between the periodic minibeam pattern and the desired lattice geometry, and is therefore particularly advantageous for small and medium‐sized tumors where conventional fixed‐pattern LRT has limited flexibility.

Because photon beams diverge from an effective source position, the multi‐slit collimator was modeled with source‐focused divergent slits rather than parallel openings. This design helped preserve the intended slit projection and reduce geometric penumbra at treatment depth.

### 2.2 Patients and treatment planning

Three brain cases and three lung cases with small and medium sized tumors were selected for this study to evaluate the proposed mini‐LRT approach. The clinical target volume (CTV) for all cases was shown in Table 1. The spatial configuration had average peak‐to‐peak distances of 12.5 mm for mini‐LRT and 25.0 mm spacing for the conventional LRT method. The lattice radiation patterns were planned using a VMAT‐like arc geometry based on discretized gantry‐angle dose influence matrices. In the present in silico planning framework, each gantry angle was represented by a precomputed dose influence matrix, and the final dose distribution was obtained by superimposing the optimized contributions from multiple angular control points and shifted collimator configurations. Therefore, the present calculation represents a planning‐level approximation of arc‐based delivery rather than a full dynamic simulation of continuous gantry rotation.

**TABLE 1:** The comparison of dose‐volume parameters between LRT and mini‐LRT for CTV and body doses for all cases.

Case	Target volume (cc)	Method	CTV peak (Gy) mean	CTV valley (Gy) mean	PVDR	Body (Gy) mean	No. of vertices	Diameter of vertices (mm)	Lattice volume ratio
1	15.75	mini‐LRT	16.13	5.73	2.82	0.26	5	4.0	1.07%
		LRT	16.44	7.96	2.07	0.38	2	15.0	22.50%
2	12.34	mini‐LRT	15.57	7.11	2.19	0.18	6	3.0	0.68%
		LRT	16.01	9.15	1.75	0.29	1	15.0	14.24%
3	28.78	mini‐LRT	15.49	6.45	2.40	0.17	9	4.0	1.05%
		LRT	16.01	9.15	1.75	0.29	2	15.0	12.31%
4	33.51	mini‐LRT	16.49	5.61	2.94	0.41	8	4.0	0.80%
		LRT	16.91	7.45	2.27	0.72	3	15.0	15.77%
5	33.21	mini‐LRT	17.10	6.20	2.76	0.43	8	4.0	0.81%
		LRT	17.10	7.31	2.34	0.70	3	15.0	15.96%
6	40.88	mini‐LRT	16.44	6.54	2.51	0.33	11	4.0	0.90%
		LRT	16.43	7.47	2.20	0.45	2	15.0	8.64%

For mini‐LRT, the vertex positions were predefined using a regular three‐dimensional lattice distribution rather than being determined during dose optimization. Specifically, candidate vertices were generated based on a hexagonal close‐packed arrangement within the CTV bounding box to achieve a dense and approximately uniform intratumoral distribution. The lattice spacing was set according to the prescribed peak‐to‐peak distance, with the corresponding step sizes defined in the left–right, anterior–posterior, and superior–inferior directions to maintain the hexagonal packing geometry. To avoid placing vertices near the target boundary, the CTV was first eroded by a margin comparable to the vertex radius, and only candidate vertices located within this eroded safe region were retained. Because the number of feasible vertices can depend on the relative alignment between the lattice grid and the patient‐specific CTV geometry, multiple translational offsets of the lattice were sampled, and the configuration accommodating the largest number of vertices within the safe region was selected. After the vertex centers were determined, spherical peak regions were generated around each vertex center, and the remaining CTV voxels were assigned as valley regions.

To accurately simulate the multi‐collimator system, detailed physical parameters were incorporated into Monte Carlo model. A custom tungsten collimator was employed, featuring a ctc spacing of 5 mm and slit width of 0.7 mm. The custom collimator was modeled with a thickness of 80 mm to ensure sufficient beam attenuation. It was positioned at a source‐to‐collimator distance of 660 mm. To minimize penumbra and ensure sharp dose gradients, the collimator slits were designed with a divergent geometry, focusing on the photon source located at the standard 1000 mm distance. Under this geometric configuration, the simulated radiation leakage through the 80 mm tungsten block was maintained at approximately 0.58 %, ensuring that unintended dose contributions to the valley regions were negligible. For a potential hardware implementation without major modification of existing linear accelerator hardware, a set of interchangeable tungsten blocks, which were made with the specific shifted slit pattern, could be manually swapped or mechanically indexed during the treatment processing.

Dose influence matrices for a 6 MV photon beam were calculated utilizing the Monte Carlo simulation platform GATE v10 (based on GEANT4 11.0).^39^ A high‐resolution dose grid of 0.5 × 0.5 × 1.0 mm^3^ was implemented to accurately capture the steep dose gradients characteristic of minibeam peak‐and‐valley distributions. The simulations employed the G4EmStandardPhysics_option3 physics list, which provides comprehensive electromagnetic physics modeling appropriate for medical physics applications. To ensure spatial accuracy within the sub‐millimeter collimator slits, production cuts for both photons and electrons were set to 0.1 mm.

### 2.3 Dose optimization

Dose optimization for mini‐LRT was formulated as a multi‐objective optimization problem that balances target coverage and OAR sparing across multiple collimator configurations. Prior to dose optimization, the predefined vertex centers were converted into spherical peak sub volumes denoted as $CTV_{\mathrm{peak}}$, and the remaining CTV voxels were assigned to the valley sub volumes denoted as $CTV_{\mathrm{valley}}$. These two subvolumes were treated as separate optimization structures. A high‐dose objective was applied to $CTV_{\mathrm{peak}}$, whereas $CTV_{\mathrm{valley}}$ was constrained to the lower dose level to maintain the intended peak‐to‐valley separation. In this study, the nominal peak and valley dose levels were 15 and 5 Gy, respectively, corresponding to a planned peak‐to‐valley dose ratio (PVDR) of approximately 3. Therefore, the optimization penalized both insufficient dose in the peak regions and excessive dose accumulation in the inter‐vertex valley regions. The resulting dose fall‐off was governed by the Monte Carlo generated minibeam dose influence matrices, which incorporated the physical collimator geometry and photon transport. After optimization, the plan was normalized by scaling the dose distribution such that $D_{95}$ of $CTV_{\mathrm{peak}}$ reached the prescribed peak dose. The final PVDR was then recalculated from the mean dose in $CTV_{\mathrm{peak}}$ and $CTV_{\mathrm{valley}}$ to verify that the spatially fractionated dose distribution was physically maintained.

The mathematical formulation was expressed as:

(1) $$\begin{array}{c} \min_{x}f\;\left(d\right)=\sum_{k=1}^{N_{k}}w_{k}\;\cdot g_{k}\left(d_{S_{k}}\right)\\ s.t.,\;\left\{\begin{array}{c} x\geq 0\\ \;d=\sum_{i=1}^{n}A_{i}x_{i}\; \end{array}\right. \end{array}$$

The objective function f(d) represented a weighted sum of penalty functions that quantified deviations from clinical objectives across all relevant structures. Each objective function $g_{k}\left(d_{S_{k}}\right)$ evaluated the dose distribution quality for structure $S_{k}$, with corresponding weight $w_{k}$ reflecting clinical priorities. The dose distribution d was computed as a linear combination of dose influence matrices $A_{i}$ and optimizable beamlet intensities $x_{i}$, where each index i corresponded to a distinct collimator configuration defining the collimator shift for mini‐LRT. The weighting coefficients $w_{k}$ were determined using an iterative objective‐balancing strategy. All weights were initially set to 1, and a preliminary optimization was performed. The magnitude of each objective‐function term was then evaluated, and the corresponding weights were adjusted to balance the relative contribution of different objectives. This procedure was repeated until the weighted objective terms were generally within one order of magnitude of each other, thereby preventing any individual objective from dominating the optimization solely due to numerical scaling.

Four types of clinical objectives were incorporated into the optimization framework to comprehensively evaluate treatment plan quality^40^, ^41^, ^42^, ^43^:

- Dose volume histogram (DVH) Maximum Constraint: This constraint limited the volume percentage receiving doses above a specified threshold. The penalty function activated when the dose to the n‐th highest ranked voxel exceeded the dose limit, ensuring that OARs did not receive excessive dose.
- DVH Minimum Dose Constraint: This constraint ensured adequate target coverage by requiring minimum volume percentages to receive prescribed dose levels. The constraint became active when the dose to the n‐th highest ranked voxel fell below the required threshold dose.
- Maximum Dose Constraint: This constraint limited the maximum allowable dose to any voxel within critical structures. All voxels exceeding the specified dose threshold were penalized, providing strict dose limitations essential for protecting radiosensitive OARs.
- Mean Dose Constraint: This constraint controlled the average dose delivered to CTV. This ensured homogeneous dose distributions within target volumes.

Equation (1) was solved using an iterative convex relaxation method^43^ with alternating direction method of multipliers (ADMM), which had been extensively utilized for solving various inverse optimization problems.^44^, ^45^, ^46^, ^47^, ^48^ The constrained problem was reformulated by introducing auxiliary variables and constructing the augmented Lagrangian:

(2) $$\begin{array}{c} \min_{x,z}\;f\left(\mathrm{Ax}\right)+\frac{\mu_{1}}{2}{\left|\left|x-z+u\right|\right|}_{2}^{2}\;\\ s.t.,\;z\geq 0. \end{array}$$

where x represented the beamlet intensity; z was introduced to handle the non‐negative dose constraints; u served as dual variable that enforces the consensus constraint x=z. The penalty parameter μ_1_ controlled the strength of the augmented Lagrangian. The optimization problem in Equation (2) was decomposed into three alternating subproblems that were solved iteratively until convergence:

- X subproblem: Beamlet Weight Optimization
(3) $$x^{k+1}=argmin\;f\left(Ax\right)+\left(\frac{\begin{array}{c} \mu_{1} \end{array}}{2}\right)\left|\right|x-z^{k}+u^{k}{\left|\right|}_{2}^{2}$$

This subproblem was reformulated as a linear system and solved using the conjugate gradient method. At each iteration, the constraint set was dynamically updated by checking voxels that violate DVH, maximum dose, or minimum dose constraints. The constraint activation mechanism sorted dose distributions within each structure and compared against clinical thresholds to determine which penalties should be enforced.

- Z subproblem: Constraint Projection
(4) $$z^{k+1}=\max\left(x^{k+1}+u^{k},0\right)$$

The z‐update performed projection onto the feasible constraint set where z ≥ 0, which enforced non‐negative dose constraints for clinical deliverability.^49^, ^50^, ^51^

- Dual Variable Update
(5) $$u^{k+1}=u^{k}+\left(x^{k+1}-\;z^{k+1}\right)$$

### 2.4 Reference SBRT treatment planning

To provide a comparison with the current standard‐of‐care approach for small and medium‐sized tumors, reference SBRT plans were generated for all lung and brain cases using conventional photon dose influence matrices. The SBRT prescription dose was 50 Gy delivered in 5 fractions, corresponding to 10 Gy per fraction. The same patient CT images and structure sets were used for the SBRT and mini‐LRT planning comparisons.

The SBRT optimization was formulated using the same inverse planning framework as described above, but with conventional photon dose influence matrices rather than minibeam dose influence matrices. Planning objectives included CTV dose coverage, CTV dose uniformity, hotspot control, external dose spillage control with 2 cm ring outside CTV, lung V_20_ reduction, spinal cord maximum dose limitation, esophagus maximum dose limitation, and D_2cm_ control. Specifically, the optimization aimed to achieve CTV D_95_ equal to the prescription dose, maintain CTV D_99_ above 90% of the prescription dose, limit the body hotspot to 125% of the prescription dose, limit external dose spillage above 105% of the prescription dose, and control D_2cm_ to 50% of the prescription dose. After optimization, each SBRT plan was normalized such that 95% of the CTV received 100% of the prescription dose. Dose‐volume metrics and RTOG‐style SBRT evaluation parameters were then calculated for all six cases. OAR constraints are shown in Table S1.

### 2.5 Robustness analysis

To evaluate the sensitivity of mini‐LRT dose distributions to spatial uncertainties, a setup‐error robustness analysis was performed for representative cases. Setup‐error dose influence matrix scenarios were generated by spatially shifting the nominal photon dose influence matrices. In addition to the nominal scenario, ten shifted scenarios were included: ± 3 mm shifts in the x, y, and z directions, and four diagonal ± 3 mm shifts in the x‐y plane. These scenarios were used to approximate translational setup uncertainties and to evaluate their impact on target coverage and spatial dose modulation.

For each setup‐error scenario, the corresponding dose influence matrix was used to recalculate the dose distribution using the same optimized beamlet intensities. Robust evaluation was performed for the predefined peak and valley structures as well as the relevant OARs. The evaluated metrics included D_95_ and D_max_ of the peak‐dose region, mean doses to relevant structures, and PVDR, which was recalculated for each scenario as the ratio of the mean peak dose to the mean valley dose.

To ensure conservative target coverage assessment, a worst‐case normalization strategy was applied. The dose distribution was scaled based on the scenario requiring the largest normalization factor for the peak‐dose region D_95_ to reach the prescribed peak dose. After this normalization, scenario‐wise D_95_, D_max_, and PVDR values were calculated and compared across all setup‐error scenarios. Representative robustness results are presented in the main text.

This robustness analysis was designed to evaluate sensitivity to translational setup uncertainty. It does not fully model dynamic delivery effects associated with continuous VMAT gantry rotation, such as gantry‐motion‐induced blurring, control‐point interpolation, dose‐rate modulation, or mechanical timing uncertainty of the shifted collimator configurations.

### 2.6 Plan evaluation metrics

Plan quality was evaluated based on target coverage, OAR doses, PVDR, conformity index (CI) and lattice volume ratio. For OAR dose evaluation, D_0.03cc_ was used as the near‐maximum dose metric instead of single‐voxel D_max_. D_0.03cc_ was defined as the minimum dose received by the hottest 0.03 cm^3^ of a given structure. The lattice volume ratio was defined as $V_{\mathrm{peak}}/V_{\mathrm{CTV}}\times 100\%$, where $V_{\mathrm{peak}}$ represents the total volume occupied by the predefined high‐dose vertices and $V_{\mathrm{CTV}}$ represents the total clinical target volume. The CI was defined according to the Radiation Therapy Oncology Group (RTOG) criteria as $CI=V_{PI}\;/V_{CTV}$, where $V_{PI}$ represents the volume encompassed by the prescription isodose and $V_{CTV}$. The R_50_ was defined as the ratio of the volume receiving at least 50% of the prescription dose to the CTV volume. The D_2cm_ was defined as the maximum dose, evaluated using D_0.03cc_, to tissue located 2 cm away from the CTV in any direction. D_x_ denotes the minimum dose received by x% of the volume, and V_x_ denotes the percentage volume receiving at least x% of the prescription dose for V100 and V90, or at least x Gy for V20.

## 3 RESULTS

### 3.1 Lattice pattern generation and geometry

**FIGURE 2:**
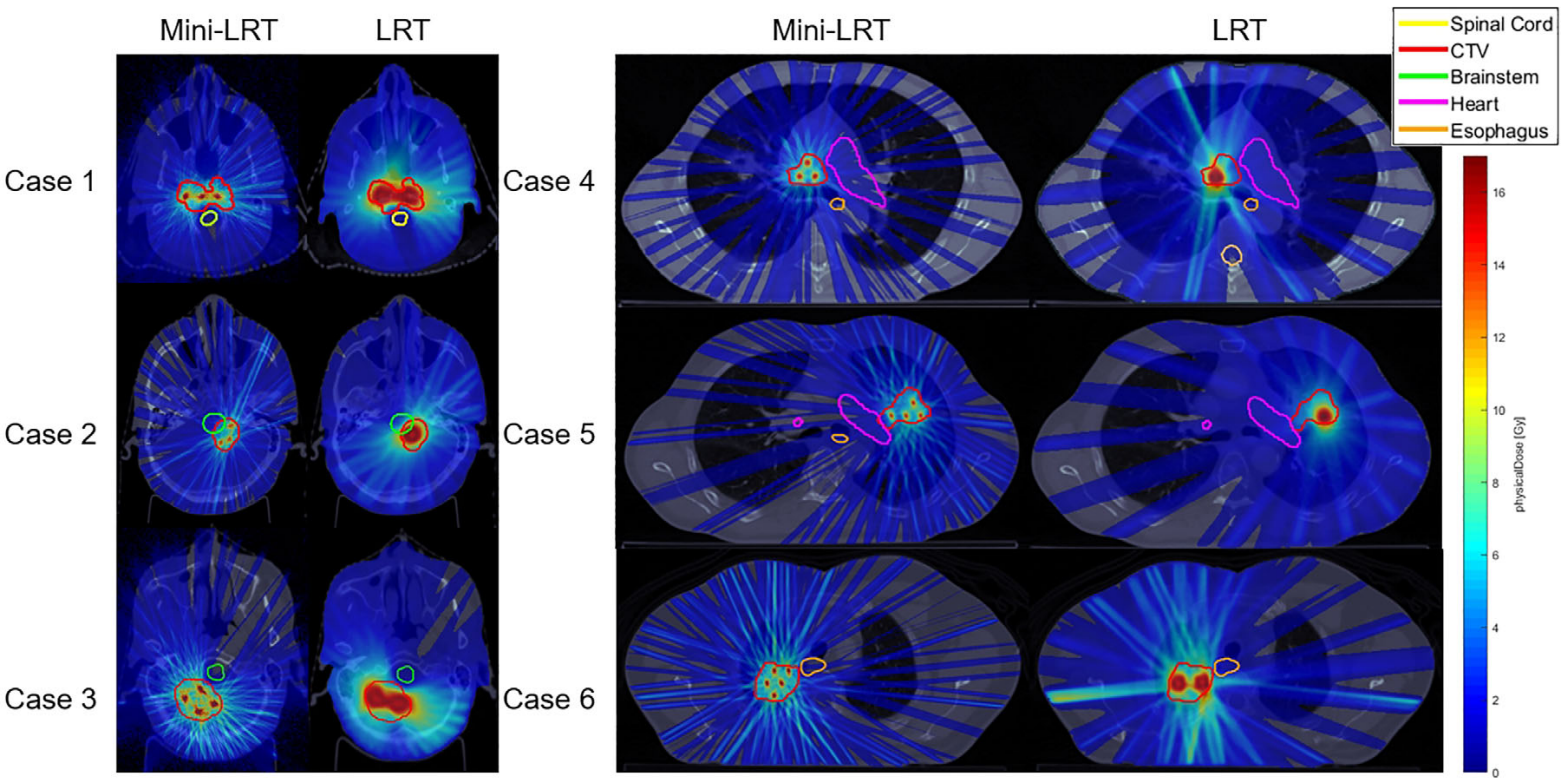
Multi‐slice dose distribution comparison between LRT and mini‐LRT. Axial dose slices for all cases.

The fundamental geometric limitations of conventional LRT become apparent when applied to medium and small tumor volumes. For the evaluated cases, conventional LRT failed to establish coherent lattice patterns within the target volumes due to its large 15.0 mm peak diameters. These large vertex dimensions relative to the tumor size resulted in geometric constraints where only 1 to 3 vertices could be accommodated within each CTV. This configuration limits the formation of a spatial lattice pattern. Multi‐slice dose distributions were shown in Figure 2.

In contrast, mini‐LRT demonstrated better geometric adaptability across all brain and lung cases. With reduced vertex dimensions (diameters of 3.0 to 4.0 mm), mini‐LRT established lattice patterns containing 5–11 vertices within the target volumes, maintaining the essential spatial fractionation characteristics. The smaller peak dimensions allowed for a higher vertex density while maintaining steep vertex‐to‐valley dose gradients.

The lattice volume ratio further quantified the relative high‐dose volume burden within the target. Despite the increased number of vertices, mini‐LRT maintained a smaller high‐dose volume fraction because of its reduced vertex diameter. Across all cases, the lattice volume ratio for mini‐LRT ranged from 0.68% to 1.07%, whereas conventional LRT ranged from 8.64% to 22.50%. This indicates that mini‐LRT increased the spatial density of vertices without expanding the absolute high‐dose occupancy within the CTV.

Furthermore, mini‐LRT achieved a higher PVDR across all cases. As detailed in Table 1, the PVDR for mini‐LRT ranged from 2.19 to 2.94, compared to 1.75–2.34 for conventional LRT. These geometric and dosimetric improvements suggest that mini‐LRT may be suitable for generating spatially fractionated dose distributions in small and medium‐sized tumors that are geometrically unsuitable for conventional LRT.

### 3.2 OAR sparing

**FIGURE 3:**
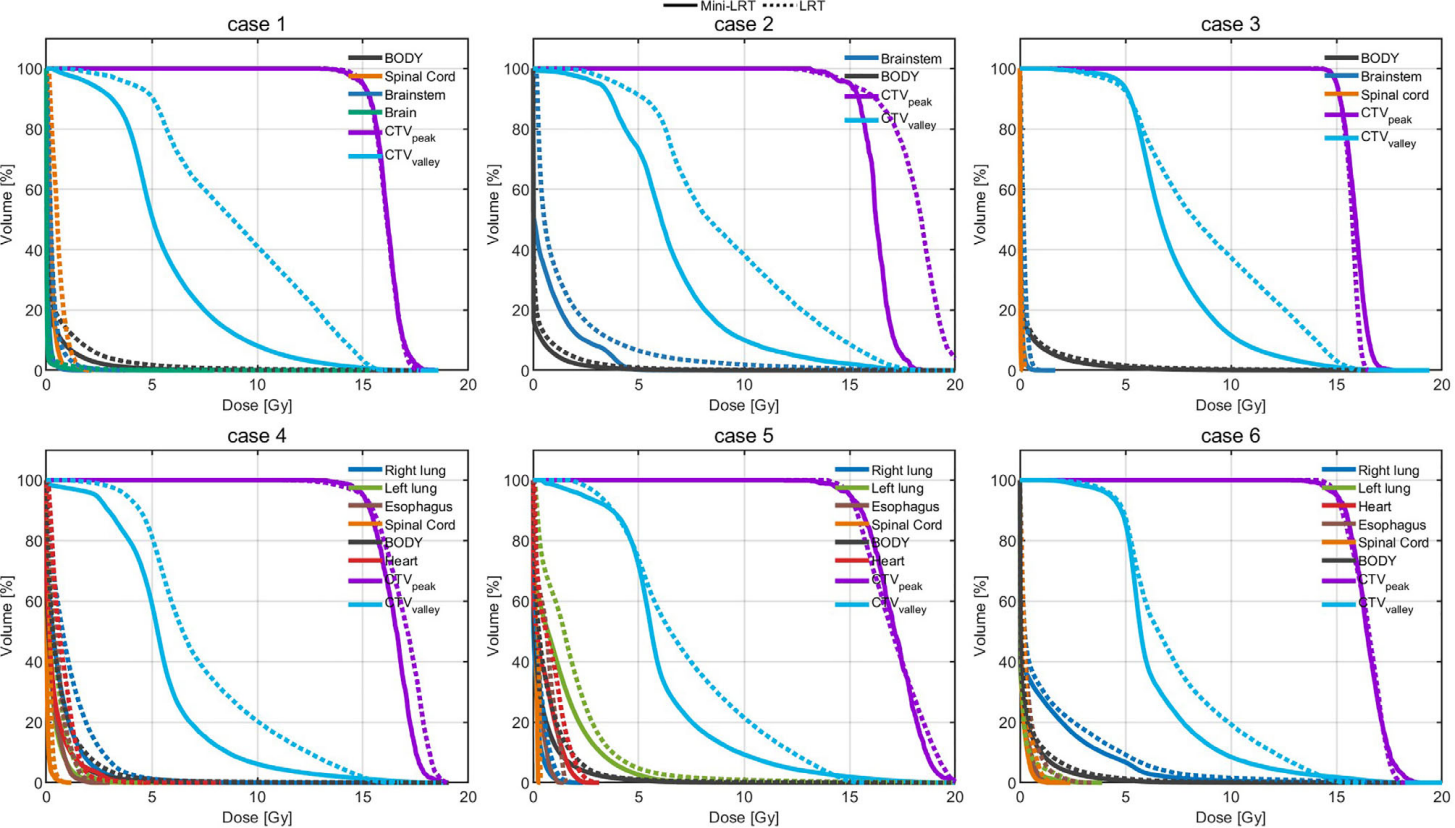
Dose‐volume histogram comparison between conventional LRT and mini‐LRT for all six cases. Solid lines represent mini‐LRT plans, and dotted lines represent conventional LRT plans. CTV peak and valley regions are shown separately.

DVH for all cases were shown in Figure 3. Tables 1, 2, 3 present the dose parameters for the target volumes and critical OARs, providing evidence of mini‐LRT's improved normal tissue sparing. While maintaining comparable target coverage, mini‐LRT exhibited lower overall dose exposure to surrounding healthy tissues. Whole‐body dose reduction was consistently observed, with mini‐LRT achieving lower mean body doses across all evaluated brain and lung cases compared to conventional LRT.

**TABLE 2:** The comparison of dose‐volume parameters between LRT and mini‐LRT for brain cases.

Brain case	Method	Brainstem (Gy) mean / D_0.03cc_	Brain (Gy) mean / D_0.03cc_	Spinal cord (Gy) D_0.03cc_
1	mini‐LRT	0.09 / 1.18	0.04 / 5.68	0.98
	LRT	0.24 / 2.83	0.06 / 15.09	1.08
2	mini‐LRT	1.16 / 6.70	/	/
	LRT	2.05 / 16.81	/	/
3	mini‐LRT	1.13 / 6.50	/	/
	LRT	2.05 / 16.29	/	/

**TABLE 3:** The comparison of dose‐volume parameters between LRT and mini‐LRT for lung cases.

Lung case	Method	Left lung (Gy) mean	Right lung (Gy) mean	Spinal cord (Gy) D_0.03cc_	Esophagus (Gy) mean / D_0.03cc_	Heart (Gy) mean / D_0.03cc_
4	mini‐LRT	0.27	0.61	1.01	0.28 / 2.90	0.40 / 4.76
	LRT	0.50	1.13	1.21	0.58 / 3.03	0.76 / 8.30
5	mini‐LRT	1.19	0.14	0.35	0.24 / 1.46	0.48 / 3.02
	LRT	1.82	0.31	0.38	0.63 / 1.60	0.80 / 3.15
6	mini‐LRT	0.13	1.06	1.34	0.14 / 1.24	/
	LRT	0.19	1.42	1.76	0.37 / 2.95	/

For brain cases, mean brainstem doses were reduced by approximately 62.50%, 43.40%, and 44.80% for Cases 1, 2, and 3, respectively. Furthermore, brainstem D_0.03cc_ was reduced. In Cases 2 and 3, brainstem D_0.03cc_ decreased from over 16.00 Gy in conventional LRT to below 6.80 Gy in mini‐LRT. Mean dose reductions were also evident in the whole brain and spinal cord.

For lung cases, the dosimetric differences of mini‐LRT were observed in the mean dose reductions across thoracic OARs. Mini‐LRT delivered lower mean doses to both lungs. Dose reductions were also achieved for centrally located organs. Compared to conventional LRT, mini‐LRT reduced the mean dose to the heart by 47.40% (Case 4) and 40.00% (Case 5). Similarly, the mean dose to the esophagus was reduced by 61.90% in Case 5. These results indicate that mini‐LRT reduces the mean dose to critical thoracic organs while maintaining the spatially fractionated high‐dose peaks within the tumor.

### 3.3 Comparison with reference SBRT plans

**FIGURE 4:**
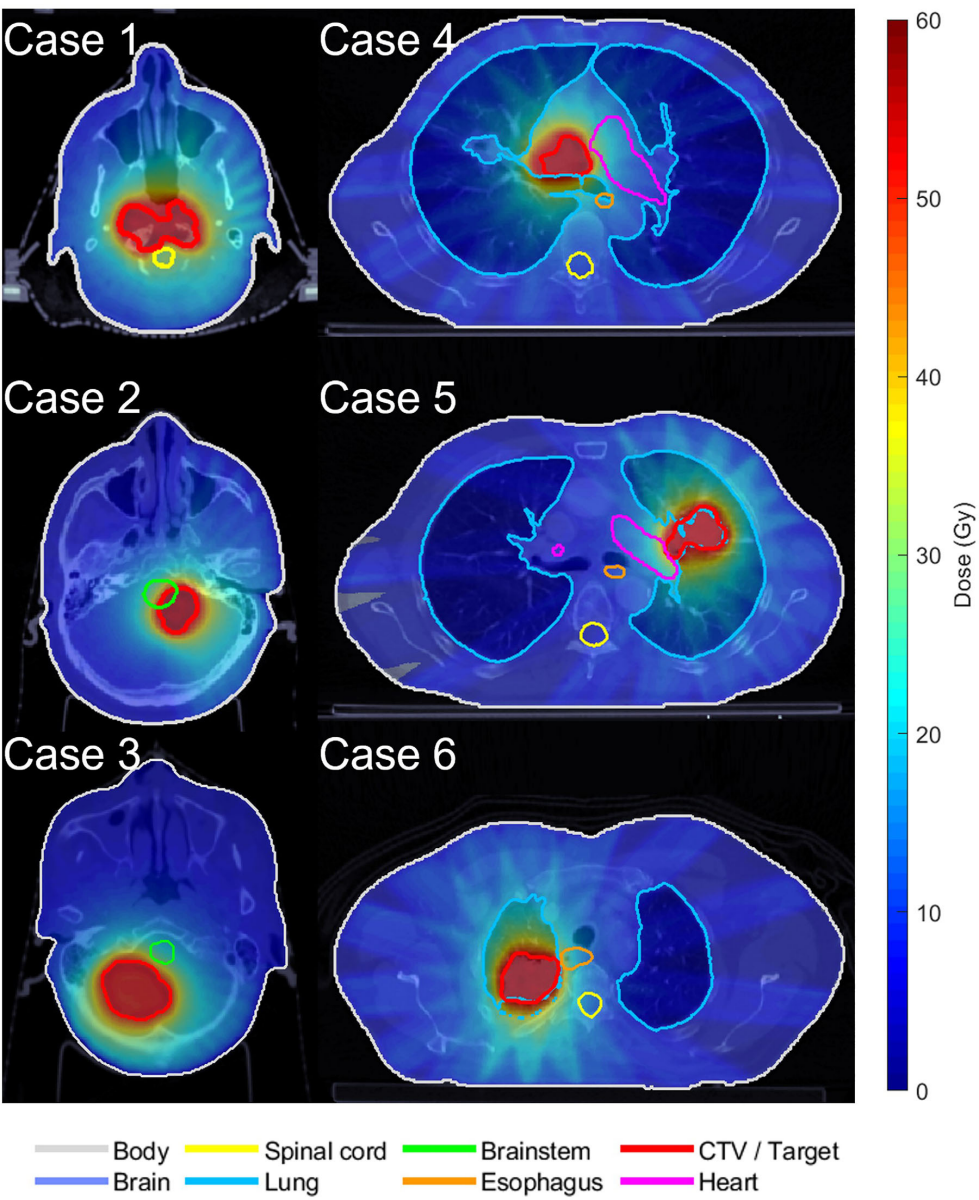
Reference SBRT dose distributions for all six cases. Axial dose distributions are shown with all plans normalized to CTV D_95_ = 50 Gy.

Reference SBRT plans were generated for all six cases to provide a standard‐of‐care comparison. Reference SBRT plan metrics are shown in Table 4. All SBRT plans were normalized to CTV D_95_ = 50 Gy. Across the six cases, CTV V_100_ was approximately 95.00%, and CTV V_90_ ranged from 98.80% to 99.04%. CTV D_99_ ranged from 43.76 to 45.09 Gy, while CTV D_0.03cc_ ranged from 55.11 to 56.81 Gy. Reference SBRT dose distributions were shown in Figure 4. DVH for all cases were shown in Figure S1. Full plan quality metrics for all SBRT plans were shown in Table S2.

**TABLE 4:** Reference SBRT plan metrics for all six cases. All SBRT plans were normalized to CTV D95 = 50 Gy.

Case	CTV D_95_ (Gy)	CTV V_100_ (%)	CTV V_90_ (%)	CTV D_0.03cc_ (Gy)	CI	R_50_	D2cm D_0.03cc_ (Gy)	Lung V_20_ (%)	OAR D_0.03cc_ (Gy)
1	50.00	95.00	99.04	56.81	1.15	5.16	28.99	/	Spinal cord: 40.37; Brainstem: 50.13
2	50.00	95.00	98.91	56.30	1.06	4.94	25.19	/	Brainstem: 51.16
3	50.00	95.00	98.80	56.75	0.99	3.75	25.83	/	Spinal cord: 3.11
4	50.00	95.00	98.89	56.18	1.11	4.72	23.99	3.74	Spinal cord: 12.20; Esophagus: 22.24; Heart: 52.72
5	50.00	95.00	98.90	56.31	1.08	4.46	27.21	10.80	Spinal cord: 8.37; Esophagus: 12.26; Heart: 48.73
6	50.00	95.00	98.90	55.11	1.02	4.08	29.10	9.38	Esophagus: 37.90

The reference SBRT plans showed generally conformal dose distributions. The CI ranged from 0.99 to 1.15, and R_50_ ranged from 3.75 to 5.16. The D_2cm_ D_0.03cc_ ranged from 23.99 to 29.10 Gy. For the three lung cases, lung V_20_ ranged from 3.74% to 10.80%.

Despite adequate target coverage, elevated near‐maximum doses to adjacent critical organs were observed in several anatomically constrained cases. In the brain cases, brainstem D_0.03cc_ reached 50.13 Gy in Case 1, 51.16 Gy in Case 2, and 33.14 Gy in Case 3. Case 1 also showed a spinal cord D_0.03cc_ of 40.37 Gy. In the lung cases, high doses were observed in organs adjacent to the CTV. Heart D_0.03cc_ reached 52.72 Gy in Case 4 and 48.73 Gy in Case 5, while esophagus D_0.03cc_ reached 37.90 Gy in Case 6. These results indicate that although reference SBRT plans achieved adequate target coverage across all cases, selected OAR‐adjacent cases still showed elevated high doses to nearby OARs. Complete OAR and objective‐function results are provided in the Supplementary Materials

### 3.4 Robustness analysis

Setup‐error robustness analysis was performed for Case 1 and Case 6, representing intracranial and thoracic cases, respectively. Representative cases metrics are shown in Table 5. Complete case‐wise results are provided in the Supplementary Materials. Full DVH envelope for all cases were shown in Figure S2. Full setup‐error robustness metrics were shown in Tables S3 and S4.

**TABLE 5:** Setup‐error robustness analysis for representative mini‐LRT cases. Worst‐case values were selected across the evaluated setup‐error scenarios.

Case	Scenario	CTV peak D_95_ (Gy)	PVDR	CTV valley D_mean_ (Gy)	CTV valley D_0.03cc_ (Gy)	OAR D_0.03cc_ (Gy)
1	Nominal	17.76	2.14	8.96	20.76	Brainstem: 1.70; Spinal cord: 1.37
	Worst‐case	14.99	2.01	9.19	20.84	Brainstem: 4.51; Spinal cord: 3.96
	Change (%)	−15.60%	−6.40%	2.60%	0.40%	Brainstem: +165.00%; Spinal cord: +188.50%
6	Nominal	16.74	1.87	9.83	21.03	Esophagus: 2.06; Spinal cord: 1.31
	Worst‐case	15.00	1.84	9.83	21.04	Esophagus: 6.05; Spinal cord: 5.55
	Change (%)	−10.40%	−1.50%	0.00%	0.10%	Esophagus: +194.40%; Spinal cord: +322.00%

For Case 1, CTV_peak_ D_95_ decreased from 17.76 Gy in the nominal scenario to 14.99 Gy in the worst case setup‐error scenario. CTV_valley_ mean dose increased slightly from 8.96 to 9.19 Gy, while CTV_valley_ D_0.03cc_ changed minimally from 20.76 to 20.84 Gy. Brainstem D_0.03cc_ increased from 1.70 to 4.51 Gy, and spinal cord D_0.03cc_ increased from 1.37 to 3.96 Gy.

For Case 6, CTV_peak_ D_95_ decreased from 16.74 Gy to 15.00 Gy. CTV_valley_ mean dose remained unchanged at 9.83 Gy, and CTV_valley_ D_0.03cc_ changed only slightly from 21.03 to 21.04 Gy. Esophagus D_0.03cc_ increased from 2.06 to 6.05 Gy. Right‐lung D_0.03cc_ remained nearly unchanged, from 20.98 to 21.00 Gy, while left‐lung D_0.03cc_ increased from 4.90 to 5.02 Gy.

The PVDR decreased from 2.14 to 2.01 in Case 1 and from 1.87 to 1.84 in Case 6 under the evaluated worst‐case setup‐error scenarios. These results indicate that spatial perturbations can reduce the sharpness of the peak‐to‐valley modulation, although the degree of PVDR degradation was modest in the tested translational scenarios.

## 4 DISCUSSION

This study demonstrates the dosimetric potential of mini‐LRT, a novel SFRT technique that addresses the geometric constraints that have historically confined conventional LRT to large tumor volumes. By employing smaller beam dimensions, mini‐LRT enables lattice patterns in tumors as small as 12.34 cc, creating smaller, more numerous, and more densely distributed high‐dose peaks within compact target geometries where conventional LRT proves geometrically infeasible. The lattice volume ratio also showed that mini‐LRT increased vertex density without increasing the high‐dose volume within the CTV, thereby maintaining a highly spatially fractionated pattern with limited high‐dose occupancy. This approach may extend the potential applicability of lattice‐based spatial fractionation to selected small and medium‐sized tumors.

Based on existing literature,^52^, ^53^ we estimate that the volume threshold separating conventional LRT from mini‐LRT applications lies approximately at 50 cc. Conventional LRT has been designed for large tumors (lesions ≥5 cm), while tumors smaller than 50 cc, experience severe geometric constraints with conventional LRT due to the inability to establish lattice patterns. Mini‐LRT's capability to treat tumors as small as 12.34 cc with 5–11 vertices enables effective treatment across a broader volume range. This extends lattice therapy eligibility to medium‐sized tumors that are too small for conventional LRT.

It is important to emphasize that mini‐LRT is not intended to replace SRS or SBRT for the general management of small and medium‐sized tumors, where standard stereotactic approaches already provide excellent outcomes. Instead, the true clinical added value of mini‐LRT lies in addressing the dosimetric dead zones encountered in extreme anatomical configurations. The reference SBRT comparison in this study supports this rationale. Although all SBRT plans achieved adequate target coverage with CTV D_95_ = 50 Gy, several OAR‐adjacent cases showed elevated near‐maximum doses to critical structures, including the brainstem, spinal cord, heart, and esophagus. These findings illustrate the dosimetric challenge of delivering uniform ablative SBRT in selected small and medium‐sized tumors located immediately adjacent to sensitive organs. In this context, mini‐LRT may provide a complementary spatially fractionated strategy rather than a replacement for SBRT. By using dense sub‐millimeter vertices and preserving low‐dose valley regions, mini‐LRT aims to maintain high intratumoral peak doses while reducing dose exposure to adjacent OARs. Therefore, its most appropriate potential application may be in carefully selected OAR‐adjacent tumors where conventional uniform‐dose escalation is constrained by normal tissue tolerance.

It should be noted that the comparison between mini‐LRT and SBRT in this study was intended as a physical dosimetric comparison rather than a radiobiological equivalence analysis. Conventional Biological Effective Dose (BED) and Equivalent Uniform Dose (EUD) models were not used as primary endpoints because mini‐LRT produces a highly heterogeneous spatially fractionated peak‐valley dose distribution, whereas standard BED/EUD models are generally based on assumptions developed for relatively uniform dose distributions. In addition, the biological response to mini‐LRT may involve bystander signaling, vascular effects, immune modulation, and differential normal‐tissue repair in the valley regions, which are not fully captured by conventional BED/EUD formulations. Future studies should incorporate radiobiological modeling, including BED/EUD or SFRT‐specific biological response models, after appropriate model assumptions are validated for mini‐LRT.

While recent innovations, such as the multi‐leaf collimator (MLC) based mini‐lattice technique proposed by Hara et al.,^36^ have made significant strides in miniaturizing spatial fractionation for clinical linear accelerators, our multi‐collimator mini‐LRT approach differs in several technical aspects that may provide potential dosimetric advantages. A primary distinction lies in the achievable spatial resolution. The MLC‐based approach is fundamentally constrained by the physical width of the MLC leaves, limiting the minimum lattice vertex size to approximately 5 mm. In contrast, the vertex size in our proposed system is dictated by the custom collimator slit width, which may enable the generation of even smaller and denser high‐dose vertices. This enhanced resolution facilitates steeper dose gradients, which may contribute to improved normal tissue sparing when treating targets adjacent to critical organs. Furthermore, the MLC‐based technique is primarily designed to deliver the peak dose; achieving a specific baseline valley dose would typically require delivering the lattice peaks followed by a separate uniform background dose. Conversely, our multi‐collimator framework co‐optimizes and delivers both the high‐dose peaks and the low‐dose valleys simultaneously within a unified treatment plan. This integrated optimization provides more explicit control over the PVDR and may improve dose coverage.

Beyond the physical dosimetric advantages, the biological rationale for reduced peak size to the mini‐LRT regime warrants discussion. The clinical efficacy of conventional SFRT is largely attributed to indirect cell kill mechanisms induced by high‐dose regions, triggering robust anti‐tumor immune responses and bystander signaling.^2^, ^9^ Because mini‐LRT is a novel concept, direct in vivo data regarding denser vertex distributions in small tumors is not yet available; however, its biological foundation can be extrapolated from both LATTICE and MBRT principles.^4^, ^5^, ^28^ Theoretically, by generating a denser array of smaller high‐dose vertices, mini‐LRT reduces the absolute physical distance between peak and valley regions within target volume. This reduced peak‐to‐peak distance may facilitate a more rapid and comprehensive diffusion of bystander signals throughout the tumor compared to the centimeter‐scale separation in conventional LATTICE. Furthermore, MBRT suggests that sub‐millimeter low‐dose valleys help preserve microvasculature and stem cells, enabling rapid normal tissue repair.^29^, ^30^ Mini‐LRT could similarly utilize this tissue‐sparing effect to protect critical organs located right next to the tumor. While our physical dosimetry supports this hypothesis, future preclinical in vivo studies are required to physically and biologically validate these tissue‐sparing benefits.

Several limitations of this study highlight opportunities for further development. First, the positions of dose peaks and their spacing were predefined rather than optimized for individual patient anatomy. A more sophisticated treatment planning system could incorporate advanced optimization algorithms to optimize peak placement, potentially positioning them in more radioresistant tumor regions while actively avoiding critical structures. Future optimization approaches could include lattice position optimization,^54^, ^55^, ^56^ collimator orientation optimization,^38^ and slit size/ctc distance optimization.^57^ Additionally, various optimization algorithms such as peak‐to‐valley dose ratio optimization,^47^ spatially‐adaptive methods,^58^ conventional mixed integer optimization approaches,^59^, ^60^ and quantum computing methods ^60^, ^61^ could be integrated to further improve plan quality and normal tissue sparing.

Second, the current methodology relies on four distinct physical collimators, which increases treatment delivery complexity and time due to multiple arc requirements and collimator changes. Future research should focus on developing dynamic collimation systems or optimized collimator designs that can achieve the necessary spatial precision without workflow interruptions, thereby improving clinical efficiency and treatment delivery practicality.^59^

Another limitation of this study is the small sample size, consisting of only six patient cases from two anatomical sites. Therefore, the present results should be interpreted as an initial proof‐of‐concept and dosimetric planning study rather than definitive evidence of broad clinical applicability. Although the selected brain and lung cases represent clinically relevant examples of small and medium‐sized tumors located near critical OARs, they cannot fully capture the anatomical variability across tumor sites, target shapes, target sizes, and tumor–OAR spatial relationships. Future studies with larger patient cohorts and more diverse disease sites are needed to evaluate the robustness, generalizability, and clinical relevance of mini‐LRT.

The setup‐error analysis provided an initial assessment of the delivery robustness of mini‐LRT. After worst‐case normalization, CTV_peak_ coverage and PVDR were reasonably maintained, suggesting that the planned peak‐valley structure was partially preserved under the evaluated translational setup‐error scenarios. The limited changes in CTV_valley_ mean dose and CTV_valley_ D_0.03cc_ further suggest that the valley‐dose regions were not markedly broadened by the simulated setup shifts. However, OAR D_0.03cc_ increased under setup perturbations, indicating that adjacent OARs remain sensitive to spatial displacement. This finding is expected because mini‐LRT relies on sub‐millimeter dose gradients, where small positional errors can shift high‐dose peak regions toward nearby OAR interfaces or low‐dose valley regions. Therefore, although the representative robustness results are encouraging, they should be interpreted as an initial setup‐error evaluation rather than a comprehensive delivery validation.

Several delivery‐related uncertainties remain to be addressed before clinical translation. The present robustness analysis was limited to translational setup‐error scenarios generated by shifting dose influence matrices and did not fully model dynamic VMAT delivery effects, including continuous gantry rotation, control‐point interpolation, dose‐rate modulation, VMAT interplay effects, mechanical collimator positioning uncertainty, respiratory motion, intra‐fraction anatomical changes, or end‐to‐end delivery accuracy. Continuous gantry rotation during beam‐on could introduce additional spatial blurring of minibeam peak positions, potentially reducing PVDR and compromising the normal tissue sparing advantages of spatial fractionation. As recent studies on respiratory motion in SFRT have demonstrated, such intra‐fraction motion significantly degrades the PVDR, with dosimetric degradation becoming more pronounced as spatial spacings decrease.^62^ This blurring effect would degrade the PVDR and potentially compromise the normal tissue sparing and biological benefits of spatial fractionation.^63^ Consequently, the clinical translation of mini‐LRT will require stringent delivery protocols, such as advanced real‐time motion management or rigid patient‐specific immobilization approaches to ensure sub‐millimeter precision.^64^ Consequently, the clinical translation of mini‐LRT will require stringent delivery protocols, such as advanced motion management or treatment delivery method ^65^ to ensure sub‐millimeter precision.

Our results confirm that the multi‐collimator approach can physically maintain these steep gradients in small to medium volumes where conventional LRT fails to generate any lattice structure. With this dosimetric framework established, experimental validation represents the critical next step.^66^, ^67^, ^68^ Preclinical studies are essential to confirm that these benefits translate into expected biological outcomes for both tumor control and normal tissue preservation. Additionally, integrating advanced image‐guidance systems could further improve the precision of peak placement, while combining this approach with FLASH radiotherapy has the potential to maximize the therapeutic benefits of this spatially refined treatment strategy.^64^, ^69^, ^70^

## 5 CONCLUSION

This proof‐of‐concept planning study demonstrates the dosimetric potential of mini‐LRT for generating spatially fractionated dose distributions in selected small and medium‐sized tumors. Compared with conventional LRT, mini‐LRT achieved smaller and denser high‐dose vertices while improving OAR sparing in the evaluated cases. Although larger and more diverse patient cohorts are required for validation, these results suggest that mini‐LRT may provide a promising strategy for further investigation in anatomically constrained small and medium‐sized tumors.

## CONFLICT OF INTEREST STATEMENT

The authors declare no conflicts of interest.

## Supporting information



SUPPORTING INFORMATION: mp70596‐sup‐0001‐SuppMat.docx
