# Supplementary material for: Photon minibeam‐based LATTICE radiotherapy for small and medium‐sized tumors: A dosimetric planning study
Source: Med Phys. 2026 Aug 3;53(8):e70596. doi: 10.1002/mp.70596 (PMC13431144; doi:10.1002/mp.70596)
Supplement: Supplementary file 1 — SUPPORTING INFORMATION: mp70596‐sup‐0001‐SuppMat.docx [file MP-53-0-s001.docx]

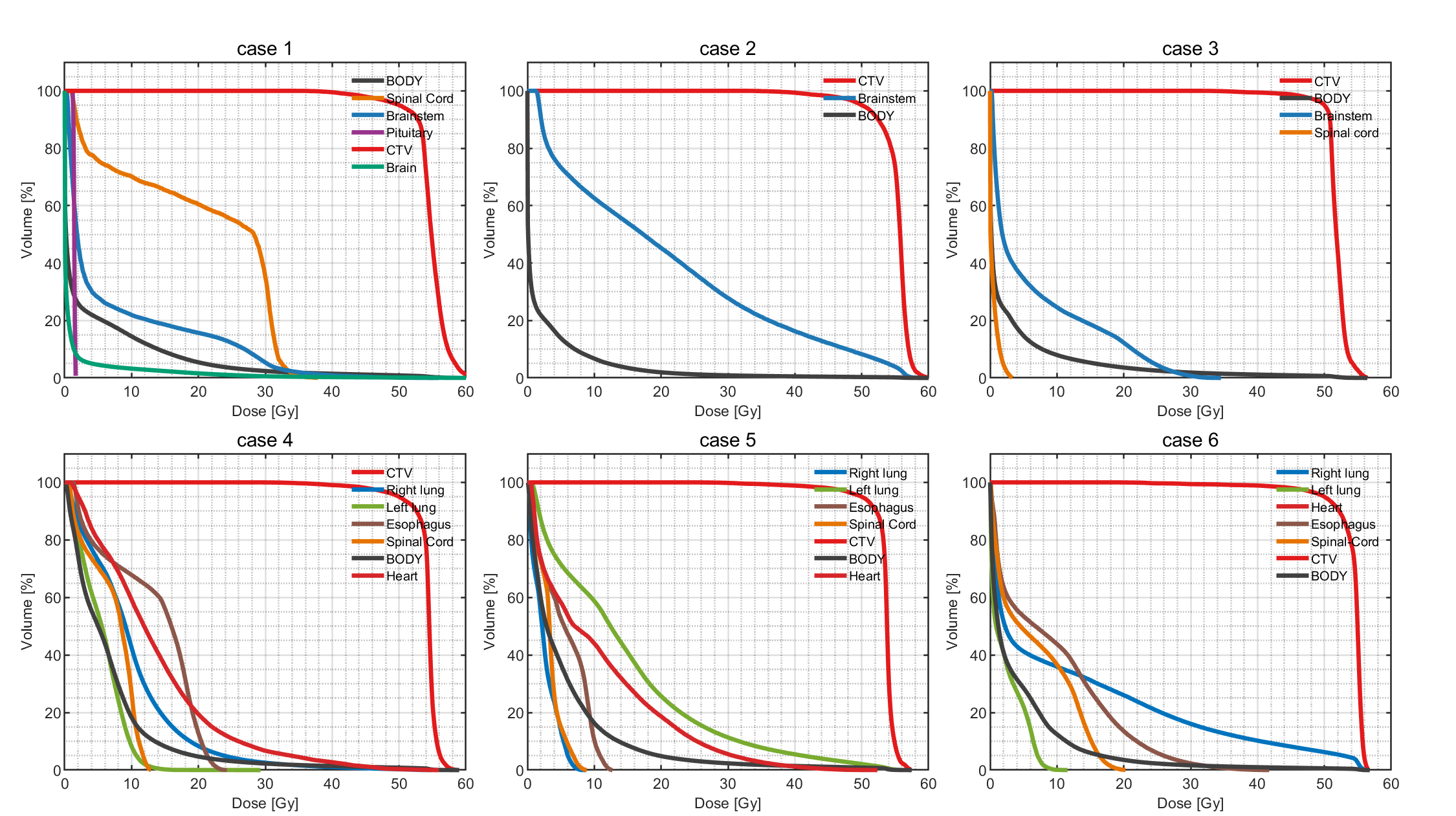


**Figure S1. DVH comparison of the reference SBRT plans for all six cases.** The three brain cases and three lung cases are shown separately. All reference SBRT plans were normalized to CTV D_95_ = 50 Gy.


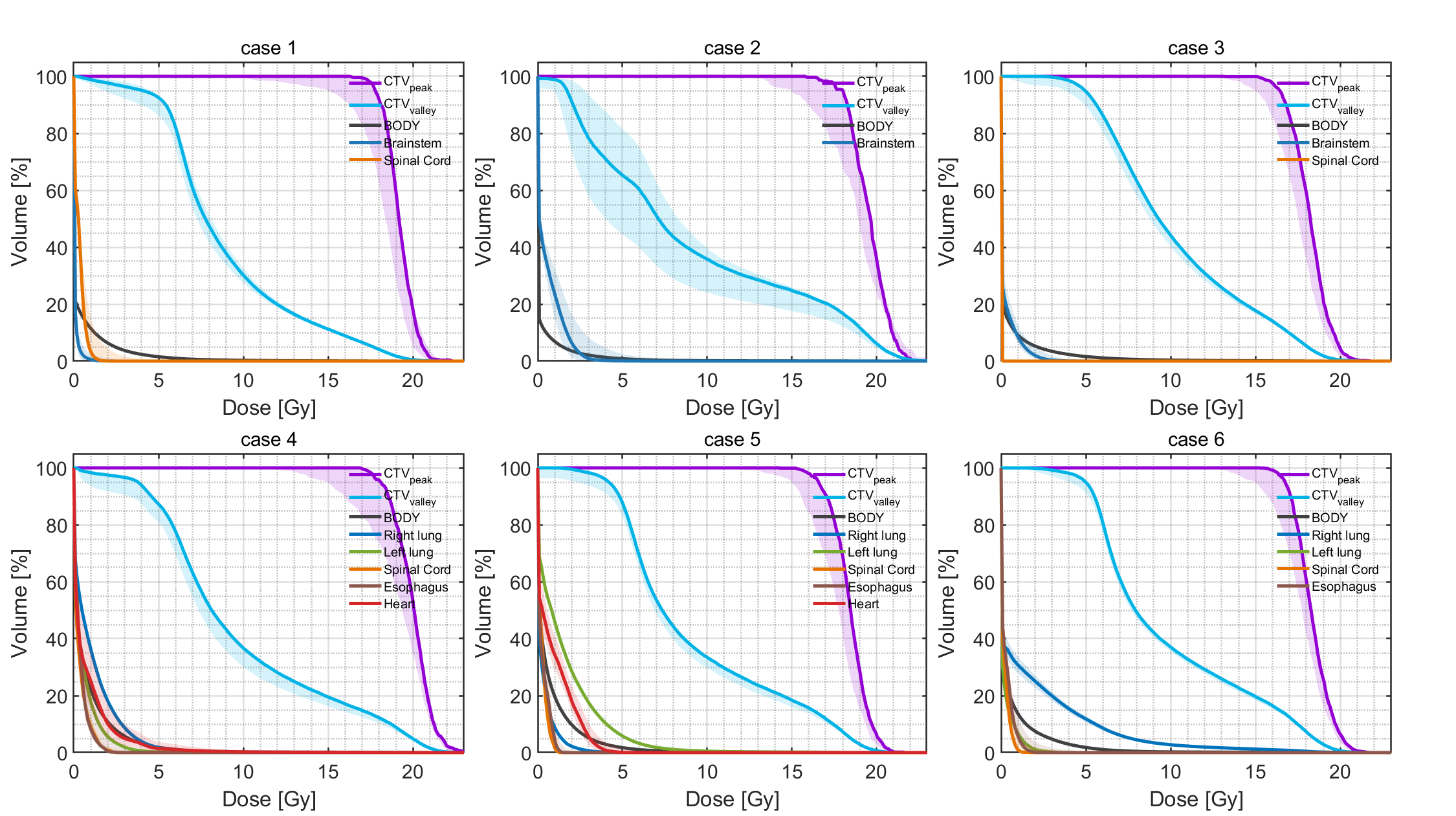


**Figure S2. Setup-error robustness DVH envelopes for mini-LRT cases.** The shifted scenarios included ±3 mm translations in the x, y, and z directions and four diagonal ±3 mm shifts in the x-y plane. The nominal DVH is shown as the reference curve. DVH envelopes are shown for CTV_peak_, CTV_valley_, and relevant OARs.

**Table S1**. Reference SBRT optimization constraint.

| **Structure / ROI** | **Objective type** | **Threshold dose (Gy)** |
| --- | --- | --- |
| CTV | Mean dose | 50.0 |
| CTV | Minimum DVH constraint | 50.0 |
| CTV | Minimum DVH constraint | 45.0 |
| CTV | Maximum dose | 62.5 |
| Body | Maximum dose | 62.5 |
| External body outside CTV | Maximum dose | 52.5 |
| 2-cm ring outside CTV | Maximum dose | 25.0 |
| Spinal cord | Maximum dose | 30.0 |
| Brainstem | Maximum dose | 30.0 |
| Lens | Maximum dose | 10.0 |
| Eye | Maximum dose | 10.0 |
| Lungs minus CTV | Maximum dose | 20.0 |
| Esophagus | Maximum dose | 35.0 |
| Heart | Maximum dose | 30.0 |

**Table S2.** Full plan-quality metrics for the reference SBRT plans.

| Case | CTV D_95_ (Gy) | CTV D_99_ (Gy) | CTV D_0.03cc_ (Gy) | CTV V_100_ (%) | CTV V_90_ (%) | CI | R_50_ | D_2cm_ D_0.03cc_ (Gy) | External D_0.03cc_ (Gy) | Lung V_20_ (%) | Lung D_0.03cc_ (Gy) | Cord D_0.03c_c (Gy) | Esophagus D_0.03cc_ (Gy) | Heart D_0.03cc_ (Gy) | |
| --- | --- | --- | --- | --- | --- | --- | --- | --- | --- | --- | --- | --- | --- | --- | --- |
| 1 | 50.00 | 45.09 | 56.81 | 95.00 | 99.04 | 1.15 | 5.16 | 28.99 | 54.09 | / | / | 40.37 | / | | / |
| 2 | 50.00 | 44.79 | 56.30 | 95.00 | 98.91 | 1.06 | 4.94 | 25.19 | 52.20 | / | / | / | / | | / |
| 3 | 50.00 | 43.76 | 56.75 | 95.00 | 98.80 | 0.99 | 3.75 | 25.83 | 52.31 | / | / | 3.11 | / | | / |
| 4 | 50.00 | 44.31 | 56.18 | 95.00 | 98.89 | 1.11 | 4.72 | 23.99 | 53.31 | 3.74 | 50.99 | 12.20 | 22.24 | | 52.72 |
| 5 | 50.00 | 44.50 | 56.31 | 95.00 | 98.90 | 1.08 | 4.46 | 27.21 | 53.28 | 10.80 | 53.20 | 8.37 | 12.26 | | 48.73 |
| 6 | 50.00 | 44.20 | 55.11 | 95.00 | 98.90 | 1.02 | 4.08 | 29.10 | 52.39 | 9.38 | 52.24 | 19.38 | 37.90 | | / |

**Table S3.1.** Full setup-error robustness metrics. Part 1.

| case | Structure | Metric | Nominal | Worst | Change (%) |
| --- | --- | --- | --- | --- | --- |
| 1 | Brain | D_0.03cc_ | 8.70 | 16.01 | 84.04 |
| 1 | Brainstem | D_0.03cc_ | 1.70 | 4.51 | 164.97 |
| 1 | CTV | D_0.03cc_ | 20.85 | 20.85 | 0.03 |
| 1 | Spinal Cord | D_0.03cc_ | 1.37 | 3.96 | 188.45 |
| 1 | CTV_peak_ | D_95_ | 17.76 | 14.99 | -15.59 |
| 1 | CTV_valley_ | D_0.03cc_ | 20.76 | 20.84 | 0.37 |
| 1 | CTV_valley_ | D_mean_ | 8.96 | 9.19 | 2.60 |
| 2 | CTV | D_0.03cc_ | 22.38 | 22.38 | 0.02 |
| 2 | CTV_peak_ | D_95_ | 18.00 | 15.00 | -16.68 |
| 2 | CTV_valley_ | D_0.03cc_ | 22.38 | 22.38 | 0.00 |
| 2 | CTV_valley_ | D_mean_ | 9.08 | 10.15 | 11.77 |
| 2 | Brainstem | D_0.03cc_ | 4.30 | 17.82 | 314.57 |
| 3 | Brainstem | D_0.03cc_ | 3.84 | 5.69 | 48.33 |
| 3 | CTV | D_0.03cc_ | 20.71 | 20.71 | 0.01 |
| 3 | Spinal Cord | D_0.03cc_ | 0.00 | 0.03 | 10033.33 |
| 3 | CTV_peak_ | D_95_ | 16.43 | 14.96 | -8.90 |
| 3 | CTV_valley_ | D_0.03cc_ | 20.69 | 20.71 | 0.10 |
| 3 | CTV_valley_ | D_mean_ | 10.29 | 10.29 | 0.00 |

**Table S3.2.** Full setup-error robustness metrics. Part 2.

| case | Structure | Metric | Nominal | Worst | Change (%) |
| --- | --- | --- | --- | --- | --- |
| 4 | CTV | D_0.03cc_ | 22.50 | 22.50 | 0.00 |
| 4 | Esophagus | D_0.03cc_ | 2.05 | 4.41 | 114.57 |
| 4 | Heart | D_0.03cc_ | 17.53 | 21.02 | 19.91 |
| 4 | Left-Lung | D_0.03cc_ | 8.21 | 8.81 | 7.35 |
| 4 | Right-Lung | D_0.03cc_ | 18.22 | 20.71 | 13.69 |
| 4 | Spinal Cord | D_0.03cc_ | 2.37 | 4.84 | 104.82 |
| 4 | CTV_peak_ | D_95_ | 18.06 | 14.94 | -17.28 |
| 4 | CTV_valley_ | D_0.03cc_ | 22.45 | 22.50 | 0.20 |
| 4 | CTV_valley_ | D_mean_ | 9.82 | 9.82 | 0.00 |
| 5 | Esophagus | D_0.03cc_ | 1.34 | 2.09 | 56.35 |
| 5 | CTV | D_0.03cc_ | 20.84 | 20.84 | 0.00 |
| 5 | Heart | D_0.03cc_ | 4.99 | 7.25 | 45.30 |
| 5 | Left-Lung | D_0.03cc_ | 19.63 | 20.42 | 4.01 |
| 5 | Right-Lung | D_0.03cc_ | 5.15 | 5.28 | 2.37 |
| 5 | Spinal Cord | D_0.03cc_ | 1.23 | 2.08 | 69.09 |
| 5 | CTV_peak_ | D_95_ | 16.59 | 15.00 | -9.62 |
| 5 | CTV_valley_ | D_0.03cc_ | 20.81 | 20.82 | 0.05 |
| 5 | CTV_valley_ | D_mean_ | 9.28 | 9.28 | 0.00 |
| 6 | Esophagus | D_0.03cc_ | 2.06 | 6.05 | 194.36 |
| 6 | CTV | D_0.03cc_ | 21.05 | 21.05 | 0.00 |
| 6 | Left-Lung | D_0.03cc_ | 4.90 | 5.02 | 2.54 |
| 6 | Right-Lung | D_0.03cc_ | 20.98 | 21.00 | 0.06 |
| 6 | Spinal Cord | D_0.03cc_ | 1.31 | 5.55 | 321.99 |
| 6 | CTV_peak_ | D_95_ | 16.74 | 15.00 | -10.39 |
| 6 | CTV_valley_ | D_0.03cc_ | 21.03 | 21.04 | 0.05 |
| 6 | CTV_valley_ | D_mean_ | 9.83 | 9.83 | 0.00 |

**Table S4.** Complete setup-error scenario-wise V_peak_ D_95_, D_0.03cc_, and PVDR.

| case | Scenario | CTV_peak_ D_95_ (Gy) | CTV_peak_ D_0.03cc_(Gy) | PVDR |  | case | Scenario | CTV_peak_ D_95_ (Gy) | CTV_peak_ D_0.03cc_(Gy) | PVDR |
| --- | --- | --- | --- | --- | --- | --- | --- | --- | --- | --- |
| 1 | nominal | 17.77 | 22.32 | 2.14 |  | 4 | nominal | 18.06 | 23.56 | 2.03 |
| 1 | x_plus_3mm | 17.31 | 22.21 | 2.11 |  | 4 | x_plus_3mm | 17.32 | 23.58 | 2.09 |
| 1 | x_minus_3mm | 16.70 | 22.64 | 2.13 |  | 4 | x_minus_3mm | 17.42 | 23.34 | 2.02 |
| 1 | y_plus_3mm | 17.16 | 22.07 | 2.11 |  | 4 | y_plus_3mm | 17.39 | 23.36 | 2.07 |
| 1 | y_minus_3mm | 17.10 | 21.56 | 2.16 |  | 4 | y_minus_3mm | 17.53 | 23.13 | 2.01 |
| 1 | z_plus_3mm | 16.54 | 21.09 | 2.01 |  | 4 | z_plus_3mm | 15.00 | 22.97 | 2.04 |
| 1 | z_minus_3mm | 16.33 | 22.32 | 2.15 |  | 4 | z_minus_3mm | 16.22 | 23.39 | 2.01 |
| 1 | x_plus_y_plus_3mm | 16.12 | 22.21 | 2.08 |  | 4 | x_plus_y_plus_3mm | 16.74 | 23.40 | 2.15 |
| 1 | x_plus_y_minus_3mm | 16.75 | 22.05 | 2.13 |  | 4 | x_plus_y_minus_3mm | 16.89 | 22.31 | 2.06 |
| 1 | x_minus_y_plus_3mm | 16.38 | 22.33 | 2.10 |  | 4 | x_minus_y_plus_3mm | 16.85 | 22.91 | 2.04 |
| 1 | x_minus_y_minus_3mm | 15.00 | 22.16 | 2.13 |  | 4 | x_minus_y_minus_3mm | 16.69 | 23.22 | 2.02 |
| 2 | nominal | 18.02 | 22.08 | 2.15 |  | 5 | nominal | 16.59 | 21.65 | 1.98 |
| 2 | x_plus_3mm | 16.44 | 22.58 | 2.41 |  | 5 | x_plus_3mm | 15.70 | 21.51 | 1.99 |
| 2 | x_minus_3mm | 17.75 | 22.75 | 2.02 |  | 5 | x_minus_3mm | 15.43 | 21.55 | 1.96 |
| 2 | y_plus_3mm | 17.46 | 22.31 | 2.41 |  | 5 | y_plus_3mm | 15.78 | 21.31 | 1.99 |
| 2 | y_minus_3mm | 17.79 | 22.26 | 1.99 |  | 5 | y_minus_3mm | 15.76 | 20.93 | 1.96 |
| 2 | z_plus_3mm | 16.77 | 22.28 | 2.32 |  | 5 | z_plus_3mm | 15.76 | 21.01 | 2.03 |
| 2 | z_minus_3mm | 17.37 | 22.60 | 2.10 |  | 5 | z_minus_3mm | 15.33 | 21.80 | 1.99 |
| 2 | x_plus_y_plus_3mm | 15.00 | 22.56 | 2.74 |  | 5 | x_plus_y_plus_3mm | 15.46 | 20.85 | 2.02 |
| 2 | x_plus_y_minus_3mm | 17.25 | 23.09 | 2.20 |  | 5 | x_plus_y_minus_3mm | 15.13 | 22.34 | 1.98 |
| 2 | x_minus_y_plus_3mm | 17.80 | 22.53 | 2.23 |  | 5 | x_minus_y_plus_3mm | 15.00 | 21.79 | 1.97 |
| 2 | x_minus_y_minus_3mm | 17.56 | 22.38 | 1.91 |  | 5 | x_minus_y_minus_3mm | 15.20 | 21.29 | 1.94 |
| 3 | nominal | 16.43 | 21.78 | 1.77 |  | 6 | nominal | 16.74 | 21.65 | 1.87 |
| 3 | x_plus_3mm | 15.85 | 21.38 | 1.77 |  | 6 | x_plus_3mm | 15.69 | 22.26 | 1.85 |
| 3 | x_minus_3mm | 15.68 | 21.67 | 1.75 |  | 6 | x_minus_3mm | 15.51 | 21.18 | 1.85 |
| 3 | y_plus_3mm | 15.90 | 21.96 | 1.78 |  | 6 | y_plus_3mm | 15.84 | 22.34 | 1.84 |
| 3 | y_minus_3mm | 15.94 | 21.59 | 1.76 |  | 6 | y_minus_3mm | 16.03 | 21.25 | 1.85 |
| 3 | z_plus_3mm | 15.14 | 20.79 | 1.75 |  | 6 | z_plus_3mm | 15.26 | 21.71 | 1.86 |
| 3 | z_minus_3mm | 15.00 | 21.30 | 1.76 |  | 6 | z_minus_3mm | 15.45 | 21.34 | 1.87 |
| 3 | x_plus_y_plus_3mm | 15.53 | 21.76 | 1.80 |  | 6 | x_plus_y_plus_3mm | 15.23 | 22.27 | 1.85 |
| 3 | x_plus_y_minus_3mm | 15.67 | 21.72 | 1.79 |  | 6 | x_plus_y_minus_3mm | 15.01 | 21.85 | 1.85 |
| 3 | x_minus_y_plus_3mm | 15.22 | 21.81 | 1.79 |  | 6 | x_minus_y_plus_3mm | 15.04 | 21.55 | 1.84 |
| 3 | x_minus_y_minus_3mm | 15.63 | 21.85 | 1.75 |  | 6 | x_minus_y_minus_3mm | 15.00 | 21.86 | 1.85 |
